# Clinicopathological and molecular features of tubo-ovarian carcinosarcomas: a series of 51 cases

**DOI:** 10.3389/fonc.2024.1427154

**Published:** 2024-08-22

**Authors:** Fan Liang, Yue Shi, Yiqing Chen, Xiang Tao, Jingxin Ding

**Affiliations:** ^1^ Department of Gynecology Oncology, Obstetrics and Gynecology Hospital of Fudan University, Shanghai, China; ^2^ Department of Pathology, Obstetrics and Gynecology Hospital of Fudan University, Shanghai, China

**Keywords:** ovarian carcinosarcomas (OCS), uterine carcinosarcoma (UCS), mutational landscape, homologous recombination deficiency (HRD) score, poly (ADP-ribose) polymerase inhibitors (PARPi)

## Abstract

**Objective:**

Tubo-ovarian carcinosarcomas are rare, extremely aggressive malignant tumors that contain both carcinomatous and sarcomatous components. Due to the disease’s rarity, developing an effective treatment strategy for ovarian carcinosarcomas has been challenging. A study was conducted to investigate the clinicopathologic and molecular features of this rare disease.

**Methods:**

We enrolled all patients diagnosed with tubo-ovarian carcinosarcomas from January 2007 to December 2022. The clinical and pathological data were gathered from medical records. Kaplan–Meier curves were plotted to calculate OS and PFS. The Log-rank test and Cox regression model were utilized to explore the relationship between clinicopathological parameters and survival. Patients with cancer tissues available had sequencing with a 242-gene panel done to investigate the mutational landscape and signature of the disease.

**Results:**

In total, 65% of the patients were diagnosed with advanced-stage cancer. The median PFS and OS of this cohort were 27 and 40 months, respectively, and there was no significant difference in survival between the homologous and heterologous components of sarcoma. Unexpectedly, staging did not have effects on prognosis. All patients had surgical attempts, and suboptimal debulking status was correlated with poorer PFS and OS. MSI was identified in 0% with low Tumor mutation burden (TMB) indicating a poor response to immunotherapy. Low HER2 expression is controversial, according to previous reports, and gives us limited choices with this rare and aggressive disease. We surprisingly found the homologous recombination deficiency (HRD)-positive status was identified in 64% of OCS, which is significantly higher than UCS and other types of epithelial ovarian cancer. The fact that all patients in our cohort who received olaparib as maintenance therapy had survived over 30 months and two had no evidence of recurrence at the latest follow-up might further validate the role of poly (ADP-ribose) polymerase inhibitors (PARPi) in the management of OCS.

**Conclusion:**

OCS patients seemed to respond to carboplatin/paclitaxel with optimal PFS and OS. Cytoreduction with no residuals proved to be the sole independent prognostic factor. WES should be done to assess the prognosis and assist with the targeted therapy, especially the HRD test, which might help select potential patients who benefit from PARPi.

## Introduction

Ovarian carcinosarcomas (OCS), also referred to as mixed malignant Müllerian tumors, are biphasic neoplasms composed of both carcinomatous and sarcomatous components. Despite accounting for 1% to 4% of ovarian malignancies, OCS are extremely aggressive and have a dismal prognosis (1). Tubal carcinosarcomas and ovarian carcinosarcomas have been analyzed and studied together due to their clinicopathologic similarities (2). Most tubo-ovarian carcinosarcomas patients were diagnosed at an advanced stage. According to statistics from the SEER Program, patients with OCS had a worse prognosis than those diagnosed with high-grade serous ovarian cancer (3).

There are usually three main hypotheses for the development of carcinosarcoma. The most widely accepted theory is the conversion hypothesis, for which the sarcomatous component could develop from its carcinomatous counterpart. The combination hypothesis states that both carcinomatous and sarcomatous elements could originate from a single progenitor cell. The last and least plausible hypothesis is the collision theory, which considers that the two components evolve independently of each other (1, 4).

Regarding the sarcomatous component, CS could be divided into homologous and heterologous subtypes (5). Homologous sarcoma could be fibrosarcoma, leiomyosarcoma, or endometrial stromal sarcoma, which is always derived from intrinsic ovarian tissue. Heterologous sarcoma usually includes bone tissue, cartilage, or striated muscles, not anything from intrinsic ovarian tissue. However, the carcinomatous component always consisted of the mono-epithelial element, and high-grade serous carcinoma is most commonly seen. Clear cell, endometrioid, squamous cell, and undifferentiated cancer may also be identified. The carcinomatous element could be composed of two or three components for less than 10% of patients (6).

Despite maximum debulking surgery followed by platinum-based chemotherapy as a conventional therapeutic option, the majority of the patients experienced recurrence within a year and survived for around 2 years (7). Compared with epithelial ovarian cancer (EOC), OCS seemed to recur earlier with a shorter survival time. New therapeutic agents are urgently required because OCS shows highly aggressive behavior and poor prognosis (3, 8).

An investigation into the mutational landscape of this rare tumor could guide us to potentially effective targeted immunotherapy.

Human epidermal growth factor receptor 2 (HER2) could enhance kinase-mediated activation of downstream signal pathways, leading to cellular hyperproliferation and malignant transformation, and its significance in the pathogenesis and subsequent target therapy in breast cancer has been consolidated. Previous studies have shown that 18%–58% of carcinosarcoma also presented with *HER2* overexpression and could potentially benefit from *HER2* target therapy (9). The estimated *HER2*/neu overexpression ranges between 25% and 56% in carcinomatous components compared to low to negligible expression in the sarcomatous element (10). One study showed HER2 overexpression in nine out of 16 cases, with gene amplification by fluorescence *in situ* hybridization (FISH) demonstrated just in one case (11). Another study revealed HER2 overexpression in nine of 28 cases, with gene amplification by FISH seen in four cases (12). *HER2* protein overexpression and gene amplification were detected in 25% (two of eight) of the primary CS cell lines (13). However, after further stratification, only one out of 13 OCS cases (7.7%) was found with positive *HER2* expression, which is significantly lower than in uterine carcinosarcoma (UCS) (12/65,18.5%), and our study seemed to hold a pessimistic result of *HER2* expression in OCS as, elaborated in the Discussion.

Mutations in BReast CAncer gene 1 (*BRCA1*), *BRCA2*, and other homologous recombination deficiency (HRD) genes may sensitize carcinosarcomas in the female genital tract to poly (ADP-ribose) polymerase inhibitors (PARPi), and the high frequency of MMR defects suggested that these tumors may also respond to immunotherapy (14). However, current studies only focused on the detection of *BRCA1/2* and other homologous recombination genes related; no investigation on the prognosis and response of PARPi based on HRD score has been reported so far.

Due to the rarity of OCS, molecular analyses have usually been done on UCS and OCS as a pool, but we gradually found these two displayed remarkably different molecular features and biological behavior (15). Molecular studies with decent sample sizes on OCS are needed to serve as more evidence for clinical management. Therefore, 51 cases of OCS diagnosed at a single institution from 2007 to 2022 were collected, analyzed, and sequenced if accessible to comprehensively present the clinicopathologic features, treatment, prognosis, and molecular characteristics of this rare disease.

## Materials and methods

### Study population

We identified patients diagnosed with OCS between January 2007 and December 2022 at our institution. All cases enrolled here were thoroughly presented and discussed on our Tumor Board, which is a weekly multidisciplinary team (MDT) meeting held in our department (Gynecology Oncology) to discuss all cancer cases of each week, and the diagnoses and management were then decided for these patients. The MDT records in Chinese were documented under each patient, which is accessible in the electronic system. We conducted a retrospective review of age, surgical extension (R0: no gross tumor, R1: residual tumor of ≤ 1 cm; suboptimal debulking surgery: residual tumor of > 1 cm), and stage as defined by the International Federation of Gynecology and Obstetrics (FIGO) in 2008 (16), perioperative chemotherapy, progression-free survival (PFS), and overall survival (OS). Pathological diagnoses of all cases were confirmed by an expert gynecological pathologist (X.T.). This study was conducted according to the Declaration of Helsinki and approved by the institutional review board of the Obstetrics and Gynecology Hospital of Fudan University (No. 2022-94). Recruited patients provided written informed consent documented.

### Statistical analysis

Survival curves were made according to the Kaplan–Meier method (17). Comparisons between survival curves were made with the log-rank statistic. The Cox regression model was employed to explore the relationship between clinicopathological parameters and survival. *p* < 0.05 was considered statistically significant. All analyses were performed with the use of IBM SPSS version 24 (SPSS Inc., Chicago, IL, USA).

### Immunohistochemical findings

The Programmed death-ligand 1 (PD-L1) IHC staining assay was performed as previously described (18). The expression of PD-L1 was assessed by IHC analysis of FFPE tumor samples using anti-PD-L1 antibodies (clone 22C3; Cat No. M3653; Dako North America, Carpinteria, CA, USA). The PD-L1 combined positive score (CPS), which is the percentage of tumor cells and surrounding immune cells showing partial or complete membrane staining, was determined and classified as negative, low positive, or high positive (CPS of < 1%, 1%–49%, and ≥ 50%, respectively) (19). The data of HER2 expression were adopted in this study. For HER2 expression, the IHC score was evaluated according to the 2016 ASCO/CAP criteria for gastric cancer (20, 21). IHC was performed using a standard FDA-approved IVD kit, the Pathway HER2 (Clone 4B5), on the BenchMark XT automated system.

(Ventana Medical Systems Inc., Tucson, AZ, USA) according to the manufacturer’s protocol. HER2 IHC score 0 is defined as HER2-null, score 1+ is defined as HER2-low, and HER2 IHC score 2+ or score 3+ is defined as HER2-high (22). Currently, no internationally accepted criteria for evaluating HER2 expression is specifically known in UCS or OCS.

### Molecular findings

Illumina next-generation sequencing (NGS) was adopted, which covered all exons, including 262 genes (listed in the **Supplementary Material**). MSI status, TMB, and HRD score were calculated based on the NGS data. Mutational signatures were extracted using base substitutions and additionally included information on the sequence context of each mutation. Furthermore, there are six classes of base substitutions: C > A, C > G, C > T, T > A, T > C, and T > G (all substitutions are referred to by the pyrimidine of the mutated Watson–Crick base pair), and information on the bases immediately 5′ and 3′ to each mutated base is incorporated in this analysis. In published studies (PNAS), applying this approach to multiple human cancer types revealed over 30 distinct validated mutational signatures. Importantly, signature 3 was strongly associated with HRD+ status within the ovarian, breast, and prostate cancer types.

## Results

**Figure 1:**
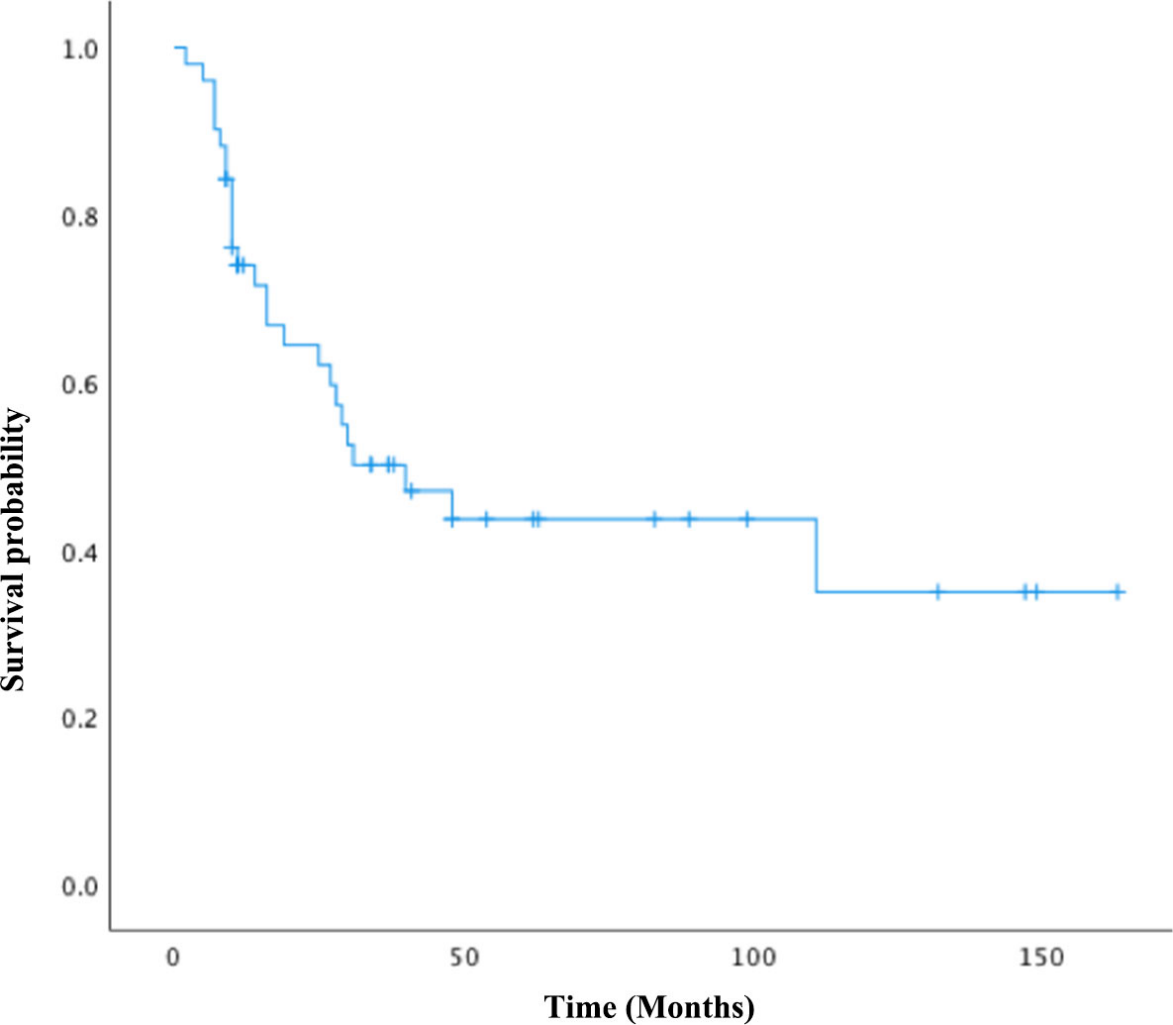
Overall survival for all patients.

In total, 51 women diagnosed with tubo-ovarian carcinosarcomas at our institution between January 2007 and December 2022 were enrolled in this study. The clinicopathologic characteristics of the cohort are summarized in **Table 1**. Four patients were found to have concurrent malignancies, including two cases of synchronous uterine carcinosarcoma, one case of cervical adenosquamous carcinoma, and another one with serous adenocarcinoma of the endometrium. The median age of onset was 59 years old, and 76.5% were postmenopausal. The average size of the tumor was 8.7 cm, with 72.5% presented as bilateral masses. The homologous subtype was identified in 28 cases (55%), and the carcinomatous element, consisting of serous carcinoma, was identified in 42 women (82.3%). Endometroid carcinoma and clear-cell carcinoma were both observed in one patient (2%), respectively. In the “mixed” carcinoma cases, four patients were diagnosed with squamous cell carcinoma, while one patient had poorly differentiated carcinoma. Heterologous differentiation was present in 23 cases (45%), chondrosarcoma in 15 patients (29.4%), rhabdomyosarcoma in four patients (7.8%), and mixed sarcoma type in four cases (7.8%). Intraoperative frozen examinations were performed in 46 cases (90.2%), all of which were suggestive of ovarian malignancy and 10 of which were straightly reported as carcinosarcoma. A total of 64.7% of the patients in the cohort had advanced cancers: 10 patients in stage I (19.6%), eight in stage II, 26 in stage III, and seven in stage IV (13.7%). Of all patients, 32 (62.7%) experienced recurrence during the study period. There were 17 platinum-sensitive, nine platinum-refractory, and six platinum-resistant patients among those who received adjuvant chemotherapy. **Figure 1** depicts the overall survival of the group. The median OS was 40 months (95% CI: 17.9–62.0 months), with the median PFS at 27 months (95% CI: 8.97–45.0 months). The 3- and 5-year OS were 38% and 19.6%, respectively.

**Table 1:** Demographic information of the study series.

Characteristics	Value
Median age at diagnosis (year)	59 (33–76)
Menopause status	
Yes	39 (76.5%)
No	12 (23.5%)
Tumor size (range) (cm)	8.7 (2.5–20)
Laterality	
Bilateral	37 (72.5%)
Unilateral	14 (27.5%)
Epithelial histology (*n*; %)	
Serous	42 (82.3%)
Endometrioid	1 (2%)
Clear-cell carcinoma	1 (2%)
> 1 carcinoma type	7 (13.7%)
Mesenchymal histology (*n*; %)	
Homologous	28 (55%)
Heterologous	23 (45%)
Chondrosarcoma	15 (29.4%)
Rhabdomyosarcoma	4 (7.8%)
> 1 sarcoma type	4 (7.8%)
Stage (FIGO)	
I	10 (19.6%)
II	8 (15.7%)
III	26 (51%)
IV	7 (13.7%)
Recurrence status	
No recurrence	19 (37.3%)
Recurrence	32 (62.7%)
Platinum sensitivity	
Platinum sensitive	17 (53.1%)
Platinum resistant	6 (18.8%)
Platinum refractory	9 (28.1%)
Median OS (range) (month)	40 (17.9–62.0)
Median PFS (range) (month)	27 (8.96–45.0)

The adjunctive therapeutic regimens are demonstrated in **Table 2**. All patients had surgical attempts, of whom 42 (82.4%) achieved optimal debulking status, with R0 (no gross tumor) in 31 cases and R1 (residual tumor of ≤ 1 cm) in 11 cases, and nine patients (17.6%) underwent suboptimal debulking surgery (residual tumor of > 1 cm). In our cohort, 48 patients (94.1%) underwent adjuvant chemotherapy, with 40 (78.4%) receiving carboplatin/paclitaxel as the first chemotherapy, two (3.9%) receiving ifosfamide/paclitaxel, three (5.9%) receiving ifosfamide/carboplatin, two (3.9%) receiving ifosfamide/adriamycin/paclitaxel (IAP), one receiving a vincristine/adriamycin/dacarbazine (VAD) regimen, and three patients refusing chemotherapy, all of whom were at advanced-stage with poor performance status. Five patients received neoadjuvant chemotherapy with carboplatin/paclitaxel (one to three cycles), and among them, four patients (80%) received optimal cytoreductive surgery (*R*0 = 2, *R*1 = 2), with one having suboptimal cytoreductive surgery.

**Table 2:** Therapeutic modalities of the 51 cases.

Characteristics	Value
Debulking surgery	
Optimal cytoreduction	42 (82.4%)
R0	31
R1	11
Suboptimal cytoreduction	9 (17.6%)
Adjuvant chemotherapy	
Carboplatin/paclitaxel	40 (78.4%)
Ifosfamide/paclitaxel	2 (3.9%)
Ifosfamide/carboplatin	3 (5.9%)
Ifosfamide/adriamycin/paclitaxel	2 (3.9%)
Vincristine/adriamycin/dacarbazine	1 (2%)
None	3 (5.9%)
Neoadjuvant chemotherapy	5
Targeted therapy	
Bevacizumab (anti-VEGF mAb)	4
hPV19 (anti-VEGF mAb)	1
Anlotinib (tyrosine kinase inhibitor)	1
Olaparib/niraparib (PARP inhibitor)	4
Trastuzumab (anti-HER2/neu)	1
Radiation	4

In our study, four advanced-stage patients received bevacizumab treatment, of whom three received bevacizumab in combination with platinum-based therapy in initial treatment and one received bevacizumab in combination with liposomal doxorubicin for platinum-resistant recurrence as second-line chemotherapy. One patient at stage IV participated in a clinical trial of hPV19 (a novel humanized antivascular endothelial growth factor [VEGF] monoclonal antibody) injection for three cycles after platinum-resistant recurrence and discontinued when disease progression was indicated by CT image, and the patient passed away with OS at10 months.

A stage IV tubal carcinosarcoma patient, who was suggested recurrence 14 months after surgery by PET-CT of a presacral mass of 7 cm in diameter, received stereotactic body radiation therapy (gamma knife treatment) and oral anlotinib (tyrosine kinase inhibitor [TKI]) treatment for 5 months as metastasis was indicated by PET-CT evaluation. One patient at stage III with HER2 overexpression was given trastuzumab in combination with platinum-based chemotherapy as initial treatment; however, liver metastasis was indicated by PET-CT follow-up with a PFS as short as 10 months. A total of four patients received PARPi in the cohort, among whom there were three cases with olaparib and one with niraparib. A stage III patient with BRCA1 mutation received olaparib maintenance therapy after optimal debulking surgery (R0) and ifosfamide/carboplatin chemotherapy. She had a survival time of 34 months with no evidence of recurrence at the last follow-up. Olaparib maintenance therapy was also employed in HRD-positive OCS patients without the BRCA1/2 mutation after optimal debulking surgery (R0) and carboplatin/paclitaxel chemotherapy, and they were all free of relapse so far. The previously mentioned patient with a presacral mass of 7 cm in diameter, who relapsed after stereotactic body radiation therapy and TKI treatment, then received secondary cytoreduction surgery followed by platinum-based chemotherapy and was maintained on olaparib for 1 year until recurrence was indicated by PET-CT. She was subsequently put back on the TC regimen and had already survived for 37 months at the latest follow-up. The three patients who received olaparib as maintenance therapy had all survived over 30 months, and two of them (66.67%) had no evidence of recurrence so far, which seems remarkably better than other target therapies, while a larger sample size is needed to consolidate the conclusion. Niraparib was used as maintenance therapy in a stage IV platinum-sensitive, recurrent OCS patient without BRCA mutations and an unknown HRD status. PARPi was discontinued when liver capsule metastasis was indicated by PET-CT scan 3 months later. Subsequently, the patient received four cycles of nab-paclitaxel every-3-week (q3w) regimen before switching to the regimen of adriamycin liposome in combination with bevacizumab, the patient died with an overall survival of 25 months.

Radiation therapy (RT) was applied to only four patients (7.8%). One patient had optimally debulking surgery followed by carboplatin/paclitaxel chemotherapy and was diagnosed with a recurrence in the peritoneum and left clavicular lymph node 14 months later; 4,500 centigray (cGy) RT was delivered to the pelvis and 6,000 cGy to the left clavicular region. The patient with an isolated recurrence of a presacral mass of 7 cm in diameter 14 months after surgery received stereotactic body radiation therapy (gamma knife therapy). The other two cases were complicated by synchronous serous adenocarcinoma of the endometrium and invasive adenosquamous carcinoma of the cervix, respectively, and were indicated for postoperative concurrent radiochemotherapy (CCRT).

**Figure 2:**
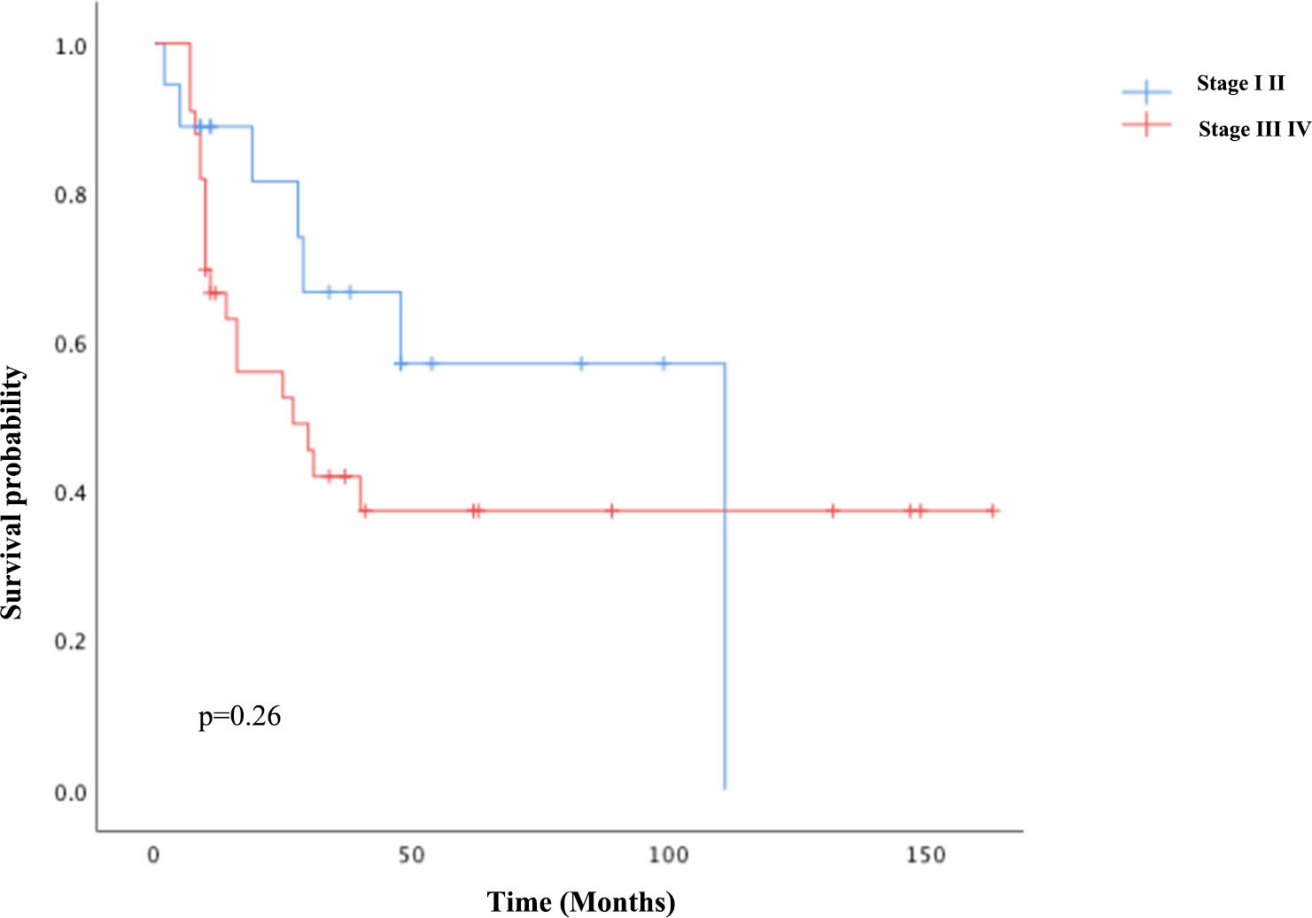
Kaplan–Meier curve of overall survival based on the stage of the disease.

**Figure 3:**
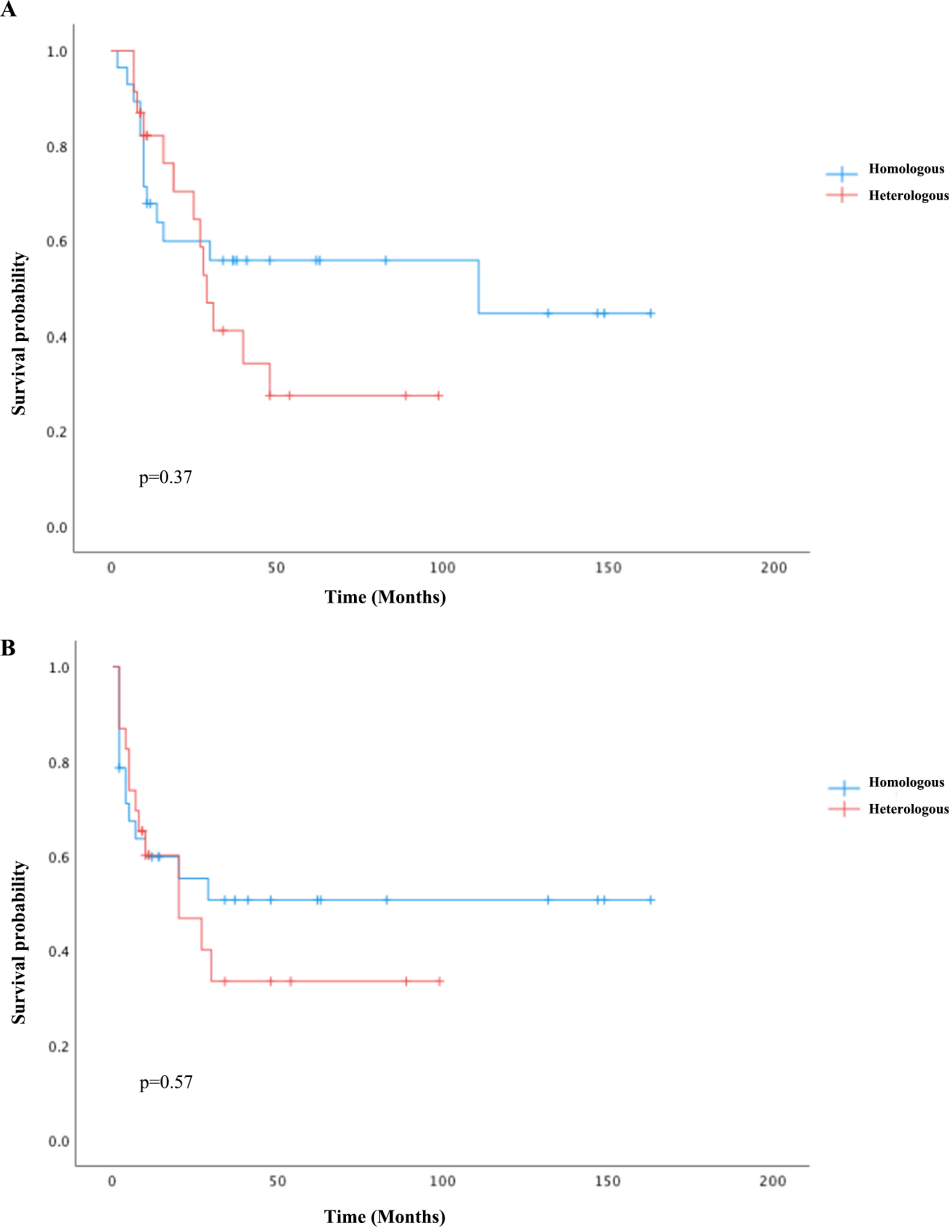
Kaplan–Meier curves comparing PFS **(A)** and OS **(B)** between the homologous and heterologous groups.

**Figure 4:**
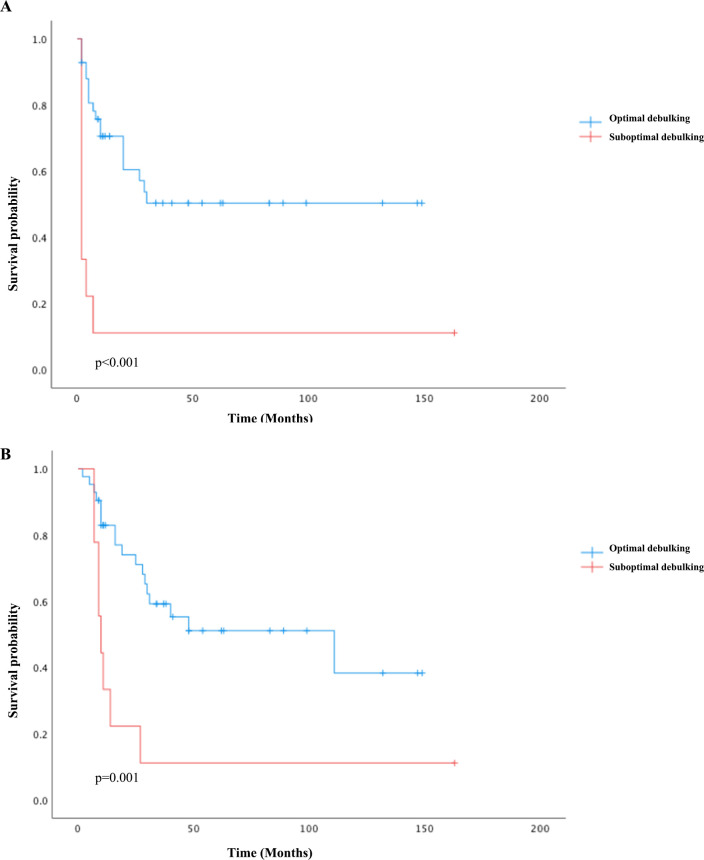
Kaplan–Meier survival curves plotted for PFS **(A)** and OS **(B)** according to debulking status: optimal debulking compared to suboptimal debulking status.

**Figure 5:**
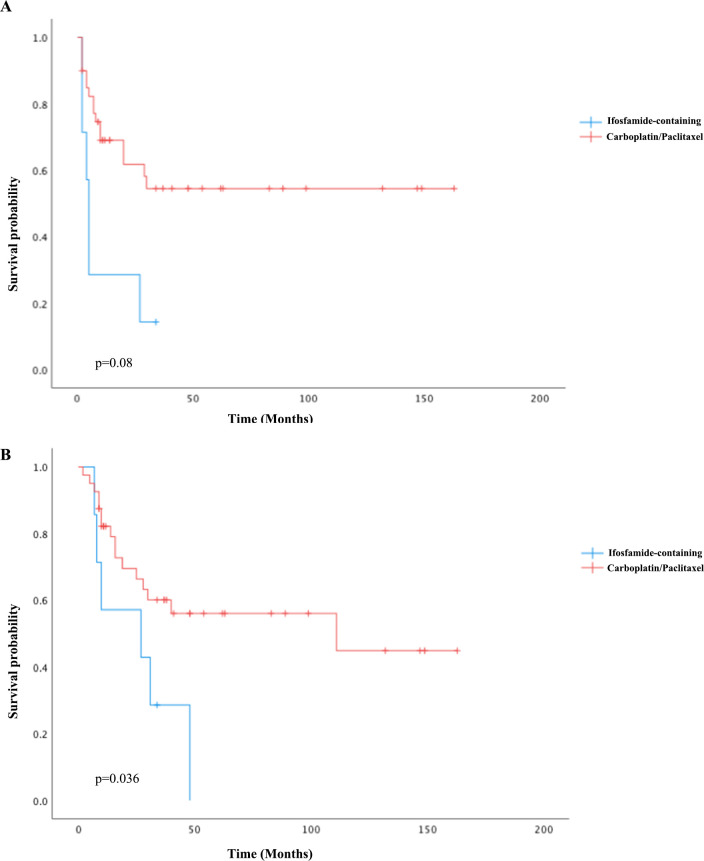
Kaplan–Meier curves of PFS **(A)** and OS **(B)** comparing carboplatin/paclitaxel to ifosfamide-containing regimens as first-line chemotherapy.

For univariate survival analysis (**Table 3**), staging of the disease might have no significant impact on OS (*p* = 0.26) (**Figure 2**). No difference was seen in PFS and OS based on histopathological subtype (*p* = 0.37; *p* = 0.57) (**Figures 3A, B**). Survival did not correlate statistically according to laterality (*p* = 0.18) or menopausal state (*p* = 0.91). However, suboptimal debulking surgery was associated with significantly worse PFS and OS (*p* < 0.05) (**Figures 4A, B**). In the subsequent multivariate analysis (**Table 4**), the Cox regression model revealed that residual disease was an independent prognostic factor (*p* = 0.008). Patients who had carboplatin/paclitaxel as first-line chemotherapy exhibited significantly prolonged PFS and OS when compared to those treated with ifosfamide-containing regimens (*p* = 0.008; *p* = 0.036) (**Figures 5A, B**).

**Table 3:** Univariate analysis of the prognosis of 51 cases of tubo-ovarian carcinosarcomas.

Variables	*n*	*p*-value
Menopause status		
Yes	39	0.91
No	12	
Laterality		
Bilateral	37	0.18
Unilateral	14	
Mesenchymal histology		
Homologous	28	0.57
Heterologous	23	
Stage (FIGO)		
I–II	18	0.26
III–IV	33	
Debulking surgery		
Optimal cytoreduction	42	0.01^*^
Suboptimal cytoreduction	9	
Adjuvant chemotherapy		
Carboplatin/paclitaxel	40	0.036^*^
Ifosfamide-containing regimens	7	

^*^p < 0.05.

**Table 4:** Multivariate analysis of Cox proportional risk model for prognosis of tubo-ovarian carcinosarcomas.

Variable in the equation						
	*B*	S.E.	*df*	Sig.	95.0% CI for EXP (*B*)	
					Lower	Upper
Debulking surgery	− 1.289	0.489	1	0.008	0.106	0.719

Variable(s) entered in step 1: adjuvant chemotherapy regimen, debulking surgery.

## Molecular findings

**Figure 6:**
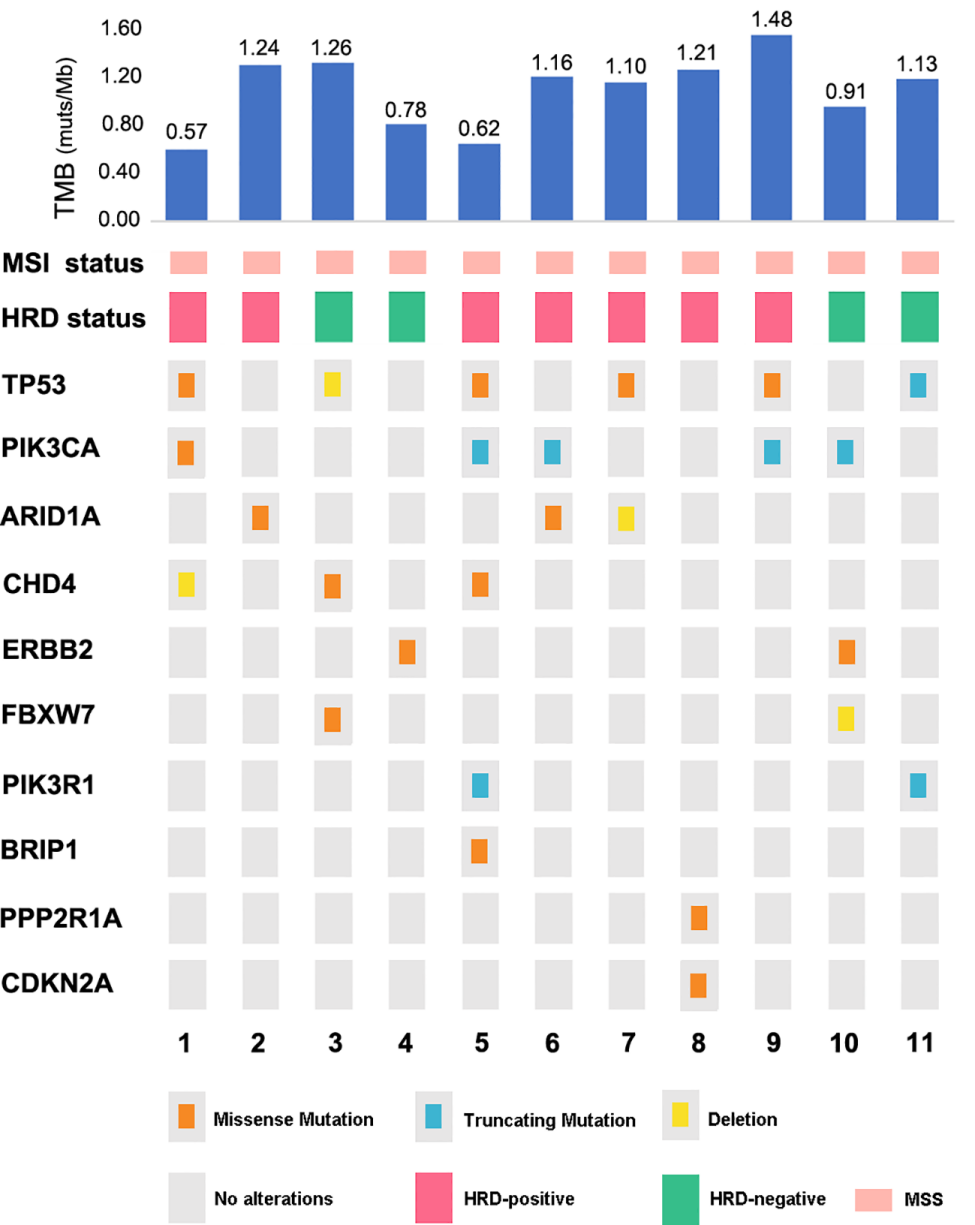
The mutational landscape across 11 OCS cases.

Tissues for the NGS test were acquired for 11 cases (11/51, 21.57%), and 242 genes associated with gynecological tumor (listed in the **Supplementary Material**), TMB, MSI, HRD score, and PD-L1 expression were comprehensively tested for the cohort. Analyses of the genetic characteristics of the somatic mutation of OCS were highly consistent with the genetic landscape results recently reported. We found alterations in multiple driver genes affecting the *ERBB2/PI3K/AKT/mTOR* pathway, the cell cycle, chromatin and remodeling pathway, and, importantly, the HRD pathways. Mutations in *TP53*, a common onco-suppressor gene, were identified in six out of 11, as demonstrated in **Figure 6**. Over half of the entire tumors had mutations in one or more of the *phosphatidylinositol 3-kinase* (*PI3K*) pathway genes: *PIK3CA* (five of 11, 45.5%), *ERBB2* (two of 11, 18.18%), or *PIK3R1* (two of 11, 18.18%), which were also frequently found in UCS. Moreover, a known recurrent mutation in *CHD4* (DNA helicase) was found in 27.27% (three of 11) of cases. Recurrent PPP2R1A mutations in uterine cancer act through a dominant-negative mechanism to promote malignant cell growth, We found one case of OCS also harboring it as a driver mutation. *FBXW7*, a known tumor suppressor gene previously identified in uterine serous carcinoma, was detected in two cases. Additionally, an HRD gene, *BRIP1* mutation, was found in one case.

Surprisingly, no phosphatase and tensin homolog deleted on chromosome 10 (*PTEN*) or Kristen rat sarcoma (*KRAS*) mutation appeared in this cohort. The most common mutation pattern was missense mutation (64%), followed by truncation (28%) and deletion. All cases proved to be MSS with a low TMB of less than 1.5 muts/Mb (median at 1.13 muts/Mb).

**Figure 7:**
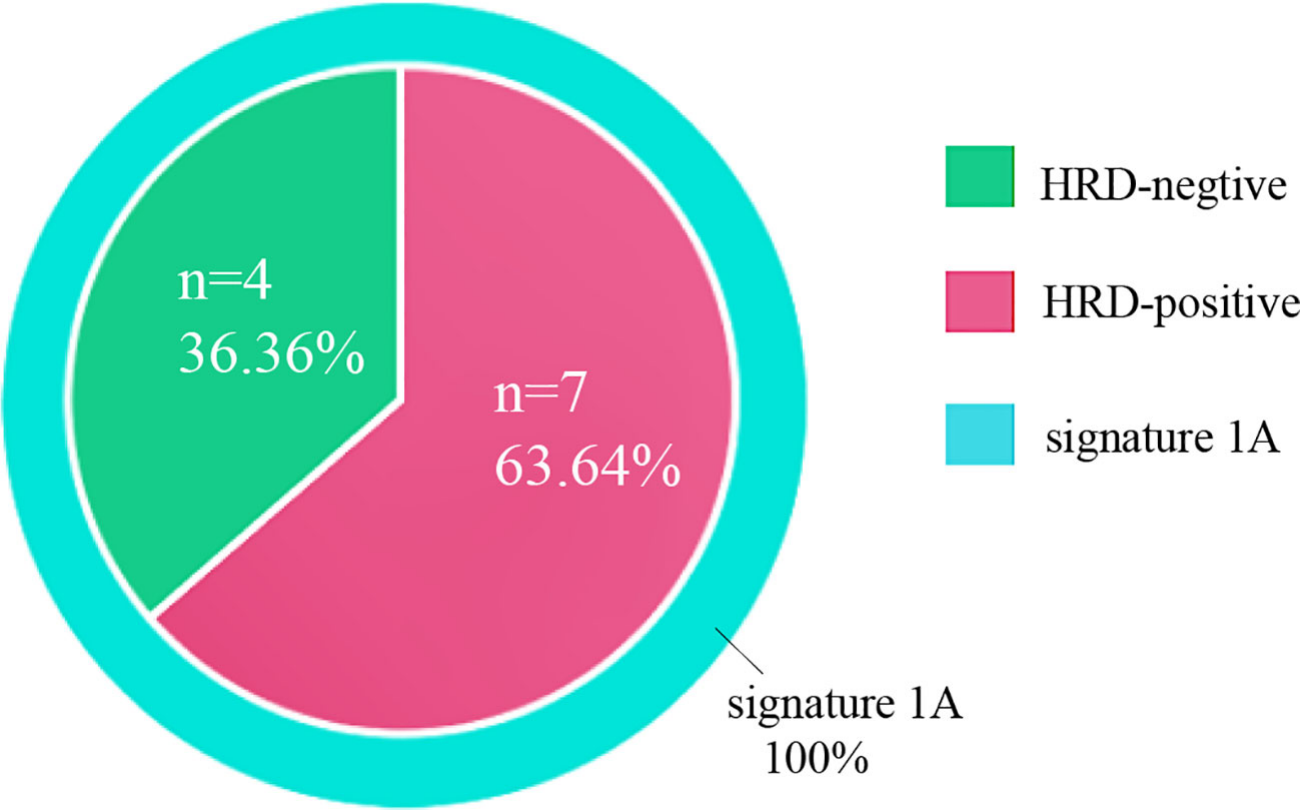
Mutational signatures of 11 ovarian carcinosarcomas. Mutational signatures of homologous recombination DNA repair defects and aging are color-coded according to the legend.

Genetic signatures of these 11 OCS cases were generated, and all cases demonstrated a dominant aging-related signature (signature 1A), as shown in **Figure 7**. HRD was calculated, and seven cases (seven of 11, 63.64%) were considered positive, taking a cutoff score of 42, which was consistent with the minor genetic signature, HRD-related signature (signature 3), distributed all over the cohort. No MSI or other signatures were detected in our cohort.

## Discussion

OCS represents a rare and aggressive gynecologic malignancy, and similar to serous ovarian cancer, patients usually presented with advanced-stage disease and were given platinum-based chemotherapy as first-line therapy (23), while patients were found to be older and have larger lesions than serous ovarian cancer (7), and also earlier recurrence and shorter survival time were seen in OCS than other pathological types of EOC.

Owing to the rarity of OCS, the prognostic risk factors have not been comprehensively elucidated. Nonetheless, several factors are known to increase the risk of OCS, including advanced age, nulliparity, decreased lactation rates, reduced use of oral contraceptives, BRCA gene mutations, and the use of assisted reproductive technologies. Poor prognostic indicators include late-stage diagnosis, older age, lymph node metastasis, suboptimal surgical cytoreduction, the presence of heterologous features in histopathology, and elevated expression of VEGF, tumor protein p53, and Wilms tumor 1 (WT1) (24). In our study, the median PFS and OS were 27 and 40 months, respectively, with 64.7% of patients presenting with advanced-stage disease, which is consistent with the cohorts reported. Optimal debulking surgery might be considered an indicator of a better prognosis (25). Our findings revealed that patients who had optimal debulking exhibited significantly better PFS and OS than those who underwent suboptimal cytoreduction. Establishing an effective adjuvant therapy for OCS has been challenging due to limited clinical evidence. In addition to optional cytoreduction, the clinical management of OCS patients largely relies on data extrapolated from other EOC or sarcomatous malignancies. It is also noteworthy that the PI3K pathway, frequently upregulated in EOC, is crucial for cell survival, chemoresistance, and the maintenance of genomic stability. Targeting the PI3K pathway in OCS may induce genomic instability and mitotic catastrophe by reducing the activity of the spindle assembly checkpoint protein Aurora kinase B, thereby increasing the occurrence of lagging chromosomes during prometaphase (26).

Given the efficacy of both cisplatin and ifosfamide, various investigations evaluated the survival benefits of platinum-taxane chemotherapy to ifosfamide-based regimens; nonetheless, the findings were inconsistent (27–29). In a phase III trial for gynecologic carcinosarcomas in 2019 (GOG-0261, NCT00954174), carboplatin/paclitaxel therapy outperformed ifosfamide/paclitaxel therapy in terms of PFS, especially in advanced-stage disease. NCCN Guidelines 2020 updated their recommendations for carboplatin/paclitaxel from an ifosfamide-based regimen based on this clinical trial (30). Our study further validated this result, as patients who underwent carboplatin/paclitaxel chemotherapy (78.4% of the cohort) had significantly prolonged PFS and OS when compared to the ifosfamide-containing regimen. About three-quarters of the patients were postmenopausal in our cohort, and there was no survival difference regarding menopausal status. It has been previously demonstrated in a study that patients with early-stage disease had greater survival benefits (31), while staging seemed insignificant for their prognosis (*p* = 0.26). The prognostic significance of the heterologous element in sarcoma has been controversial. Some prior studies supported the idea that homologous subtypes might benefit survival (32, 33), while our study showed no difference in survival regarding this categorization in sarcoma histology.

Unlike UCS, RT was seldom used in the treatment of early-stage OCS patients (34). RT was considered beneficial only in the management of isolated pelvic recurrences for OCS (1). In this case, only four (7.8%) patients received RT in our study, and the potential impact on prognosis could not be generated due to the limited number.

Comprehensive genetic landscape studies of ovarian and uterine CS have recently unequivocally demonstrated that the carcinomatous and sarcomatous elements of these rare but highly aggressive tumors derive from a common precursor having mutations typical of carcinomas (35). Consistent with this view, *TP53* mutation was identified as a dominant driver mutation in OCS (54.5%), with the highest mutation frequency, while still significantly lower than that in UCS (62%–91%) and serous ovarian carcinoma (36, 37).

Although the role of *TP53* as a dominant driver mutation in OCS has been established, the mutation frequency in our cohort seemed higher than in the previous study, at about 30% (15). Mutations in *PIK3CA* and *ARID1A* were more seen in OCS with an endometrioid epithelial component, and *TP53*, *CHD4*, and *FBXW7* mutations were more frequently found in CS with a serous high-grade epithelial component, which is in accordance with previous studies in UCS. However, a larger sample size is definitely needed to validate this conclusion (38, 39). Interestingly, mutations in *KRAS* and *PTEN* were not detected in our OCS cohort, which were mutations commonly found in UCS.

As *HER2* positivity was recommended to be defined as 2+ and 3+ scores on protein expression for gynecologic carcinosarcomas, only two patients (two of 11, 18.2%) met the criteria, which is significantly lower than what was reported in UCS while remarkably higher than that in serous ovarian carcinoma (9).

The diverse genetic patterns among OCS, UCS, and serous ovarian carcinosarcoma were also displayed in their genetic signature features. Mutational signatures have recently provided major insight into the biological processes shaping the tumor genome and can potentially inform therapeutic modalities in multiple tumor types (40). Our cohort demonstrated all cases with a dominant aging-related signature (signature-1), plus seven cases (63.64%) also showing HRD-related signatures (signature-3), which were consistent with their HRD scores. Although no *BRCA* mutation was detected, HRR genes such as *BRIP1* mutation were revealed, and a high incidence of *TP53* mutation could greatly contribute to the genetic scarring and HRD status. This positivity is even higher than the HRD status in serous ovarian carcinoma at 50% (41). HRD patterns in OCS were seldomly analyzed, and our results concluded for the first time that WES-extracted mutational signature consistent with HRD score is significantly more commonly found in OCS (63.64%) when compared to UCS (15%–25%). Aging was identified in 100% of OCS in our cohort, which was close to 86% reported in UCS. In general, OCS presented its tumor signature in line with ovarian cancer, mainly as signature 1 (aging) mixed with signature 3 (HRD).

With these diverse features taken together, it should be noticed that although OCS and UCS may look histologically similar, they significantly differ in their molecular features. Our results may support a differential biological behavior between OCS and UCS, more like serous ovarian cancer, which indicated higher sensitivity to platinum-based chemotherapy and PARP inhibitor than UCS. Importantly, potentially broader use of PARPi might result in improved prognosis in OCS. Pennington et al. (30) also found that four of 12 OCS patients demonstrated loss-of-function mutations in HRR genes, suggesting that PARP inhibitors might be beneficial in this circumstance.

Four out of seven HRD+ patients in our cohort received olaparib as maintenance therapy; all had a survival time of more than 30 months, and two of them had no evidence of recurrence at the latest follow-up. MSS with a low tumor mutation burden was found in all cases, which also differed from the 13%–22% of UCS patients who presented as MSI. This could be explained by the fact that about 85%–90% of UCS had endometrioid histology, which much less happened in OCS. The median TMB of 1.13 muts/Mb is similar to the TMB for ovarian cancer reported (38). Osamu et al. utilized TCGA endometrial molecular stratification system in 92 UCS and 17 OCS samples. OCS comprises mostly CNH tumors with low TMB, while UCS is more heterogeneous in genomic aberration subtypes, and a better prognosis with high TMB was seen, especially in *POLE* and MSI-H groups (42). As in serous ovarian cancer (43, 44), therapy targeting the EGF family and VEGF may be effective in the treatment of OCS. Zorzou et al. (45) reported in their study that four (44%) of nine OCS patients expressed VEGF, a biomarker associated with poorer prognosis in various cancers, and antiangiogenic agents like bevacizumab could be considered potentially useful (46, 47). hPV19 mAb, a novel humanized monoclonal antibody against VEGF by binding to VEGF-R1 and VEGF-R2, was used in one case in our cohort while the disease progressed under the medication. A prospective clinical study with a larger sample size is needed to validate the VEGF inhibitor’s use in the treatment of OCS. Anlotinib, a novel small-molecule tyrosine kinase inhibitor (TKI), targets multiple tyrosine kinases, including vascular endothelial growth factor receptor (VEGFR), fibroblast growth factor receptor (FGFR), platelet-derived growth factor receptors (PDGFR), and c-kit. Cui et al. (48) demonstrated that anlotinib exhibited moderate improvements in PFS and OS in platinum-resistant and platinum-refractory ovarian cancer. It suggested that anlotinib might be an alternative therapeutic option for platinum-resistant or platinum-refractory ovarian malignancies. One patient in our cohort used anlotinib, and the therapeutic effect of TKI in OCS needs further study.

In our study, the patient with *HER2* overexpression who received trastuzumab in combination with platinum-based chemotherapy was found to have liver metastasis with a PFS of 10 months. Further studies are warranted to explore whether HER2-positive OCS patients may benefit from trastuzumab treatment. Trastuzumab emtansine (T-DM1) was proved to be highly efficient in HER2-positive OCS cells in a mouse model (13). In a phase I clinical study (NCT02277717), a novel HER2-targeting ADC (SYD985) has exhibited promising benefits in locally advanced and metastatic tumor cells, indicating that further investigation in OCS patients is warranted. While in our cohort, only two cases displayed OCS *HER2* overexpression, the utilization of HER2 inhibitors could be limited due to the low rate.

The role of immune checkpoint inhibition in carcinosarcoma remains obscure. Although the expression of PD-L1 has been evaluated in a wide range of cancers, there is limited information available in tubo-ovarian carcinosarcomas. In a study of OCS, PD-L1 overexpression was found in 53% of carcinomatous components and 47% of sarcomatous components (49), which was inversely correlated with survival. Of our cohort, 54.5% showed positive PD-L1 expression (CPS ≥1), while no one was put on an immune checkpoint inhibitor as a clinical trial for OCS with PD-1 was unavailable.

Given the high positivity of HRD and PD-L1 expression, a synergistic effect of PARP inhibitors and anti-PD1 therapy could be anticipated to be effective. Additionally, BRCA deficiency has been shown to trigger a STING-dependent innate immune response, leading to the production of type I interferons and proinflammatory cytokines. Moreover, studies have indicated that PARP inhibition can deactivate GSK3 and upregulate PD-L1 in a dose-dependent manner, subsequently suppressing T-cell activation and enhancing cancer cell apoptosis, which might make this combination of target therapies potentially efficient in OCS patients (50). The ROCSAN trial (NCT03651206), which began in 2020, will investigate the effectiveness of niraparib (PARPi) and dostarlimab (PD-1 inhibitor) in gynecological CS, and the data are keenly awaited.

It is hard to draw a conclusion about the potential impact of PARPi in the management of OCS due to the limited number of cases receiving target therapy in our study. However, our results add to the existing understanding and clinical evidence for treating this rare malignancy. Further studies with collaborative, prospective multi-institutional trials are needed to help elaborate the molecular features and therapeutic strategy for patients with this rare and aggressive disease.

## Data Availability

The datasets presented in this study can be found in online repositories. The names of the repository/repositories and accession number(s) can be found in the article/**Supplementary Material**.
