# Supplementary figures and images for: Clinicopathological and molecular features of tubo-ovarian carcinosarcomas: a series of 51 cases
Source: Front Oncol. 2024 Aug 22;14:1427154. doi: 10.3389/fonc.2024.1427154 (PMC11375614; doi:10.3389/fonc.2024.1427154)

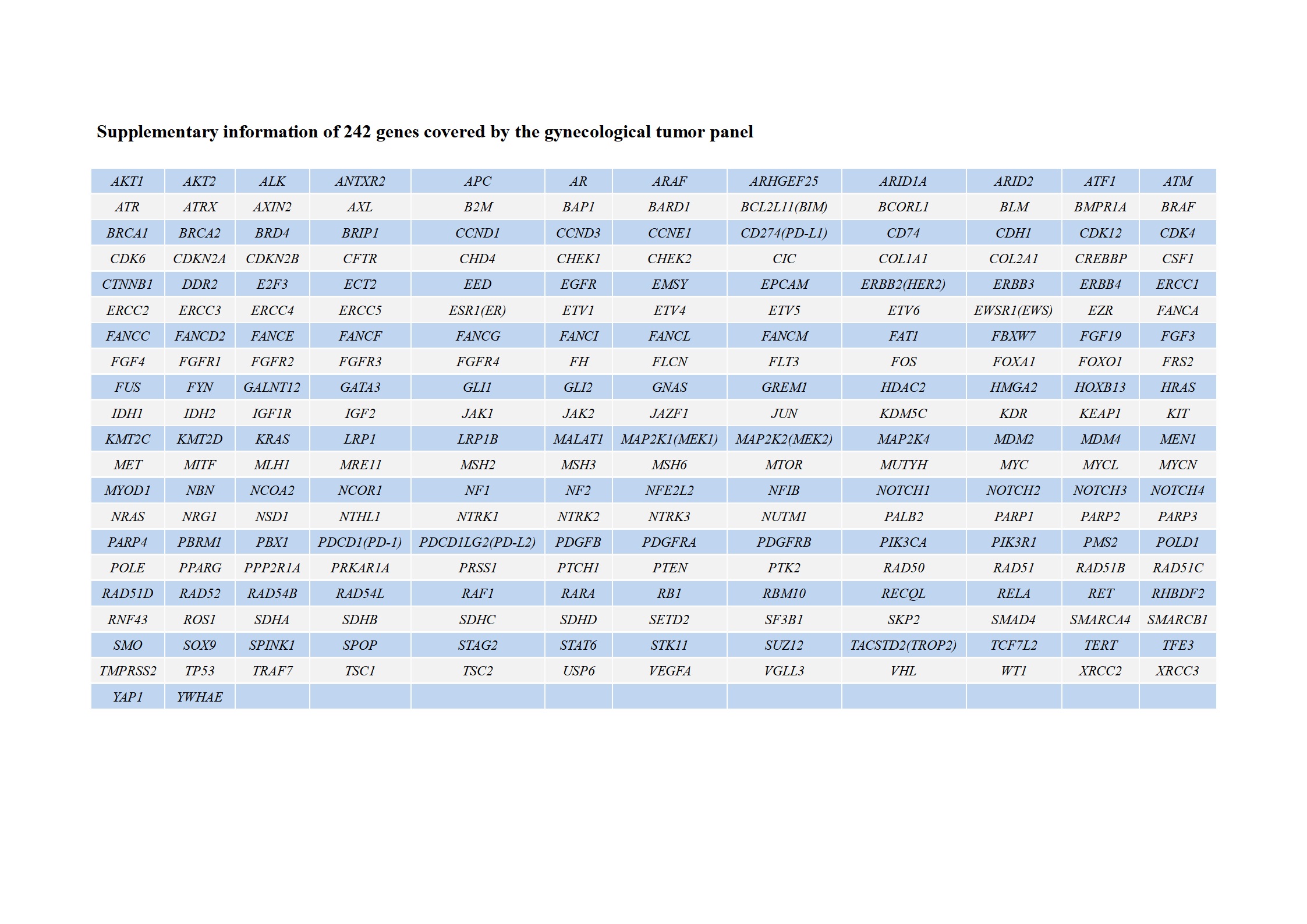

Supplement: Supplementary file 1 [file Image1.jpeg]
